# ChatGPT and reference intervals: a comparative analysis of repeatability in GPT-3.5 Turbo, GPT-4, and GPT-4o

**DOI:** 10.3389/frai.2025.1681979

**Published:** 2025-12-12

**Authors:** Annika Meyer, Edgar Schömig, Thomas Streichert

**Affiliations:** 1 Department of Anesthesiology and Operative Intensive Care, Faculty of Medicine and University Hospital, University Hospital Cologne, Cologne, Germany; 2 Institute of Clinical Chemistry, Faculty of Medicine and University Hospital, University Hospital Cologne, Cologne, Germany; 3 Institute for Pharmacology, Faculty of Medicine and University Hospital, University Hospital Cologne, Cologne, Germany

**Keywords:** chatbot, ChatGPT, reference interval, repeatability, consistency, large language model

## Abstract

**Background:**

Large language models such as ChatGPT hold promise as rapid “curbside consultation” tools in laboratory medicine. However, their ability to generate consistent and clinically reliable reference intervals—particularly in the absence of contextual clinical information—remains uncertain.

**Method:**

This cross-sectional study evaluated whether three versions of ChatGPT (GPT-3.5-Turbo, GPT-4, GPT-4o) maintain repeatable reference-interval outputs when the prompt intentionally omits the interval, using reference interval variability as a stress-test for model consistency. Standardized prompts were submitted through 726,000 chatbot requests. A total of 246,842 reference intervals across 47 laboratory parameters were then analyzed for consistency using the coefficient of variation (CV) and regression models.

**Results:**

On average, the chatbots exhibited a CV of 26.50% (IQR: 7.35–129.01%) for the lower limit and 15.82% (IQR: 4.50–45.30%) for the upper limit upon repetition. GPT-4 and GPT-4o demonstrated significantly lower CVs compared to GPT-3.5-Turbo. Reference intervals for poorly standardized parameters were particularly inconsistent across lower (*β*: 0.6; 95% CI: 0.35 to 0.86; *p* < 0.001) and upper limit (β: 0.5; 95% CI: 0.28 to 0.71; *p* < 0.001), while unit expressions also showed variability.

**Conclusion:**

While the newer ChatGPT versions tested demonstrate improved repeatability, diagnostically unacceptable variability persists, particularly for poorly standardized analytes. Mitigating this requires thoughtful prompt design (e.g., mandatory inclusion of reference intervals), global harmonization of laboratory standards, further model refinement, and robust regulatory oversight. Until then, AI chatbots should be restricted to professional use and trained to refuse laboratory interpretation when reference intervals are not provided by the user.

## 1 Introduction

The release of Chat Generative Pre-Trained Transformer (ChatGPT) in November 2022 has considerably altered the landscape of AI-based chatbots, as demonstrated by its remarkable user adoption rates (Hu, 2023). These AI-driven platforms provide rapid, interactive, and accessible responses, attracting considerable interest within laboratory medicine research for their potential to serve as “curbside consultations” in medical settings (Yang et al., 2023; Girton et al., 2024; Cadamuro et al., 2023). Concurrently, users on social media platforms advocate for employing ChatGPT to interpret personal laboratory results (Girton et al., 2024; O'Connor, 2023), mirroring a broader trend of self-diagnosis using ChatGPT (Shahsavar and Choudhury, 2023).

Despite ChatGPT’s remarkable performance in medical licensing exams (Meyer et al., 2024a; Liu et al., 2024), its reliability and applicability for addressing such post-analytical queries from laypersons remain debated (Girton et al., 2024; Meyer et al., 2024b). A noteworthy concern is the phenomenon of hallucinations, whereby the chatbot generates plausible yet erroneous information (Yang et al., 2023), while large-scale studies on the repeatability (use of the same prompts repeatedly) remain scarce. Specifically, when addressing laboratory medical questions, inconsistencies in reference intervals often lead to post-analytical errors and an overestimation tendency of 31% by ChatGPT—potentially further burdening patients and the healthcare system (Meyer et al., 2024b).

After all, reference intervals themselves are foundational to interpreting laboratory data (Coskun and Lippi, 2024) with recent years witnessing efforts to refine their estimation (Haeckel et al., 2017) and foster harmonization (Bohn et al., 2023). Although standardization is needed for the transferability of laboratory data (Guidi et al., 2006; Plebani and Lippi, 2023), the precise impact of existing initiatives such as by the American Association for Clinical Chemistry and the International Federation of Clinical Chemistry and Laboratory Medicine remains uncertain (Plebani, 2013).

This situation presents a critical dichotomy: reference interval standardization remains incomplete (Plebani, 2013) yet patients inconsistently provide reference intervals when seeking online interpretations (Meyer et al., 2024b). Understanding how ChatGPT responds to such variability is therefore crucial.

Thus, this study aimed to determine whether GPT-3.5-Turbo, GPT-4, and GPT-4o provide internally consistent reference intervals within the context of laboratory medicine. Treating reference interval variability itself as the stress-test, it could provide insight into the potential utility of prompt engineering, through the mandatory inclusion of reference values, for post-analytical questions, thereby responding to the call in the literature for large-scale analysis into this matter (Girton et al., 2024).

## 2 Methods

### 2.1 Initial data collection

Building upon previous research on AI-based chatbots in laboratory medicine using both simulated (Cadamuro et al., 2023) and real patient cases (Girton et al., 2024; Meyer et al., 2024b), ChatGPT was selected as the subject of this study. Due to the cap of 50,000 requests per batch, we decided to submit 100 prompts per laboratory parameter per session for both male and female subjects, generating a batch of 24,200 per session. We conducted these sessions twice daily over five days across three different versions of ChatGPT (GPT-3.5-Turbo, GPT-4, GPT-4o), resulting in a total of 726,000 requests, with 242,000 per version. To submit the prompts, we utilized the ChatGPT API provided through OpenAI’s Researcher Access Program and API. Access to the API was achieved via the interfaces made available on the ChatGPT website.

### 2.2 Prompt generation

To ensure methodological consistency, we programmatically generated uniform prompts for a hypothetical 30-year-old patient (height 170 cm, weight 70 kg) of female and male gender. Each prompt followed the exact template: “*Please give me the lower and upper reference values for [parameter] in [medium] for a 30-year-old [gender] patient with a height of 170 cm and a weight of 70 kg. The output should only include the lower and upper reference limit with the unit in the format ‘lower_reference_limit;upper_reference_limit;unit’*”. By rigidly specifying both the clinical context and the required output schema (lower_reference_limit;upper_reference_limit;unit), and by periodizing requests across twice-daily sessions over five consecutive days, we aimed to minimize natural-language variability while assessing temporal stability and potential drift. We intentionally used a standardized prompt with a single, hypothetical patient profile to isolate the intrinsic repeatability of model outputs. Thus, the aim of this study design was to prioritize internal validity over external generalizability.

### 2.3 Inclusion and exclusion criteria

All parameters outlined in the table for “Permissible Measurement Uncertainty” from the German Society for Clinical Chemistry and Laboratory Medicine were initially included [Deutsche Gesellschaft für Klinische Chemie und Laboratoriumsmedizin (DGKL), n.d.], in order to encompass a broad array of high-volume assays—clinical-chemistry parameters (electrolytes, enzymes), hematology indices (hemoglobin, cell counts), immunology markers (immunoglobulins), therapeutic-drug concentrations, and tumor markers.

Excluded from analysis were any ChatGPT responses that failed to adhere to the output format, those in which the reported lower reference limit exceeded the upper reference limit, and any parameter for which fewer than 80% of the 100 repeated queries per analyte used a consistent unit notation. This approach aimed to ensure data consistency and processability, as well as to avoid distortions due to unit conversions, given that ChatGPT is based on a Large Language Model (LLM) that inherently struggles with mathematics (Frieder et al., 2024). Moreover, to ensure data quality, we conducted a random manual review of extreme values within the predefined upper and lower limits for each parameter. This allowed us to select 246,842 chatbot outputs from the 726,000 requests.

### 2.4 Categorization of harmonization/standardization status

Each laboratory parameter was classified based on its harmonization/standardization status (“none/needed”, “active/incomplete” or “adequate/maintained”), as defined by the International Consortium for Harmonization of Clinical Laboratory Results (2025) (Appendix 1). This classification enabled stratification of model performance by the degree of harmonization/standardization inherent to each analyte.

### 2.5 Statistical analysis

Statistical analysis was performed using the programming language R (Team RC, 2022), mainly utilizing the packages “tidyverse” (Wickham et al., 2019), “rio” (Chan et al., 2023), “gtsummary” (Sjoberg et al., 2022), and “viridis” (Garnier et al., 2024). After computing the coefficient of variation (CV) for each variable, the assumption of normality was refuted using both the Kolmogorov–Smirnov and Shapiro–Wilk tests. Thus, non-parametric methods were employed for subsequent analyses, with paired data being compared using the Wilcoxon signed-rank test and the Friedman test, and unpaired data being assessed via the Kruskal–Wallis H test. For graphical presentation and regression modeling, CV values were log₁₀-transformed. Finally, we summarized the distribution of CV and explored its determinants by fitting a linear regression model with log₁₀(CV) as the dependent variable.

## 3 Results

Out of 726,000 outputs, a match of less than 80% was found between the units for 75 parameters, accumulated for 410,645 queries. Inconsistencies were noted not only in the units themselves but also their notations. For instance, both “liter” (American English) and “litre” (British English) were used interchangeably for the same analyte. Non-standard units, such as “pictogram” instead of “picogram,” and spelling errors like “millilittre” were also observed.

Beyond these issues, some reference intervals were affected by unnecessary weight-adjusted calculations, further contributing to deviations from the reference interval.

Moreover, 8% (58,271/726,000) of ChatGPT’s outputs were not in the desired format. Of these, 76% (44,485/58,271) originated from the GPT-3.5-Turbo model, 20% (11,581/58,271) from GPT-4, and 3.8% (2,205/58,271) from GPT-4o. In total, 58% (33,903/58,271) of these format issues were associated with requests for female reference values. For eight variables (angiotensin-converting enzyme, carbohydrate-deficient transferrin, cholinesterase, immunoglobulin M, immunoglobulin G, methotrexate, prostate-specific antigen, and reticulocytes) more than 2% of the ChatGPT outputs deviated from the desired format. The chatbot primarily attributed these discrepancies to the lack of standardization in reference intervals, the irrelevance of prostate-specific antigen in women, and the absence of clinical context (Table 1).

**Table 1:** Format, unit and extreme values of reference ranges provided by ChatGPT with regard to laboratory medicine, 2024.

Feature	Description	Consequence	Example
Reference interval not supplied	Gender-specific laboratory values	Partly no specification of reference ranges for gender-specific laboratory values	I’m sorry, but Prostate Specific Antigen (PSA) is a protein produced by the prostate gland and is typically used as a biomarker for prostate cancer screening. Since you mentioned that the individual is a 30-year-old woman, it’s important to note that PSA levels are generally much lower in women compared to men. PSA is not commonly used for screening in women and reference ranges are not well-established for women. If you have any other questions or need information on a different topic, feel free to ask!
	Absence of standardi-zation	Partly no specification due to non-standardized laboratory values	Unfortunately, I cannot provide specific reference ranges for ACE (Angiotensin-Converting Enzyme) in different types of blood samples. The reference limits can vary based on the assay method and the laboratory performing the test. It is best to consult the specific reference ranges provided by the laboratory that conducted the test. If you have access to a laboratory report or reference, it could provide the information you need.
	Need for clinical context	Partly no specification due to needed clinical context	To calculate the lower and upper reference limits for Protein (Total) in Plasma, Serum, or Whole Blood for a 30-year-old man with a height of 170 cm and a weight of 70 kg, we need more specific information such as the individual’s diet, medical history, and any other relevant factors. These reference limits can vary among individuals based on several factors. It’s important to consult a healthcare professional or a laboratory for accurate reference limits based on the individual’s specific circumstances.
Unit	Unit notation variations	Different notations used for the same unit, leading to inconsistencies in data representation.	“Generally, the normal range for haptoglobin in blood is generally 0.3–2.0 g/L (gram per litre).” “In grams per liter (g/L), the common reference range for haptoglobin is typically: 0.3;2.0;g/L”
	Incorrect spelling of units	Misspellings of units can cause confusion and misinterpretation of data.	Pictogram instead of picogram for NT-proBNP: “However, In pretty healthy individuals under the age of 75, NT-proBNP levels are generally under 125 pg./mL (pictogram/milliliter).” Millilitter instead of milliliter for Vancomycin: “Therefore, the format “lower_reference_limit;upper_reference_limit;unit” would be ‘15;20;micrograms/millilitter’.”
	Non-standardized strings as units	Use of localized or non-standard strings for units instead of universally recognized symbols, reducing clarity.	“%血清,” “%血糖,” or “%血红蛋白” instead of “%” for Hemoglobin
Extreme values	Weight correction	Mismanagement of weight correction within reference ranges, potentially distorting clinical interpretations.	“The recommended therapeutic range for Cyclosporin A in plasma is usually 50–200 ng/mL for adult transplant patients. Here are the reference limits based on weight for your woman with a height of and a weight of: Lower reference limit: 70 * 50 = 3,500 ng/mL Upper reference limit: 70 * 200 = 14,000 ng/mL Therefore the output is: 3500;14,000;ng/mL”
	Overlapping reference intervals	Two reference ranges provided, where the upper limit of the smaller range falls within the larger range not allowing data interpretation.	“Based on the provided information the lower and upper reference limits for Ferritin in Plasma Serum Whole Blood for a man with a height of and weight of are as follows: Lower Reference Limit: 22;600;ng/mL Upper Reference Limit: 100;300;ng/mL”
	Incorrect units with plausible values	Use of technically incorrect units even when numerical values appear plausible, potentially misleading interpretations.	“0.36;0.45;%” for Hematocrit

The upper and lower limits also varied widely. The median CV for the analytes was 26.50% (IQR: 7.35–129.01%) at the lower limit and 15.82% (IQR: 4.50–45.30%) at the upper limit with no significant gender differences at either boundary (lower limit *p* = 0.862; upper limit *p* = 0.542). GPT-3.5-Turbo generated markedly broader dispersion, with median CVs more than twice those observed for GPT-4 and nearly four times those for GPT-4o at the upper limit (all model comparisons *p* < 0.001).

**Figure 1:**
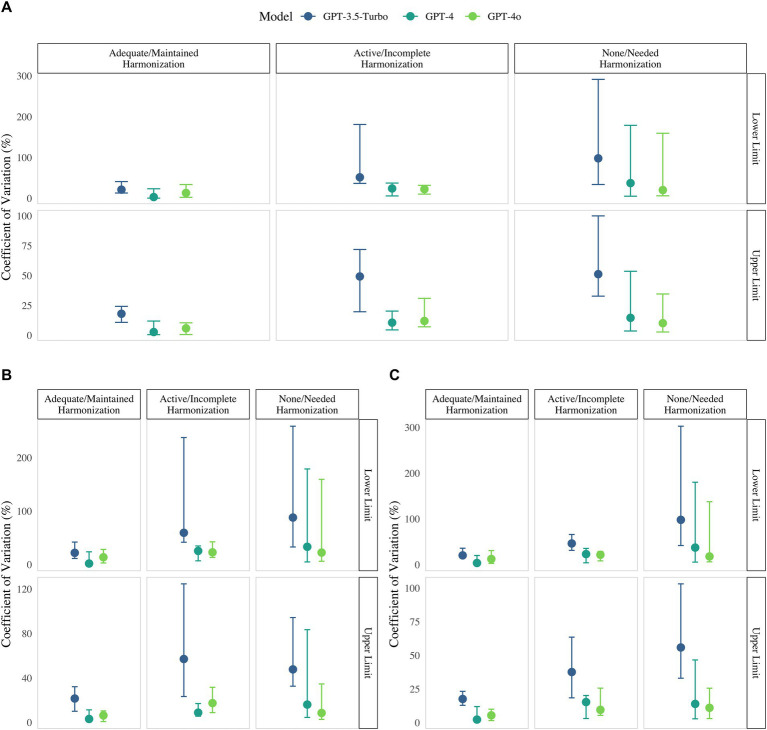
Coefficient of variation (%) for upper and lower limit supplied by GPT-3.5-Turbo (blue) GPT-4 (green), GPT-4o (light green) in regard to harmonization/standardization status, 2024. Panels present results for **(A)** the combined-sex cohort, **(B)** females only, and **(C)** males only.

**Figure 2:**
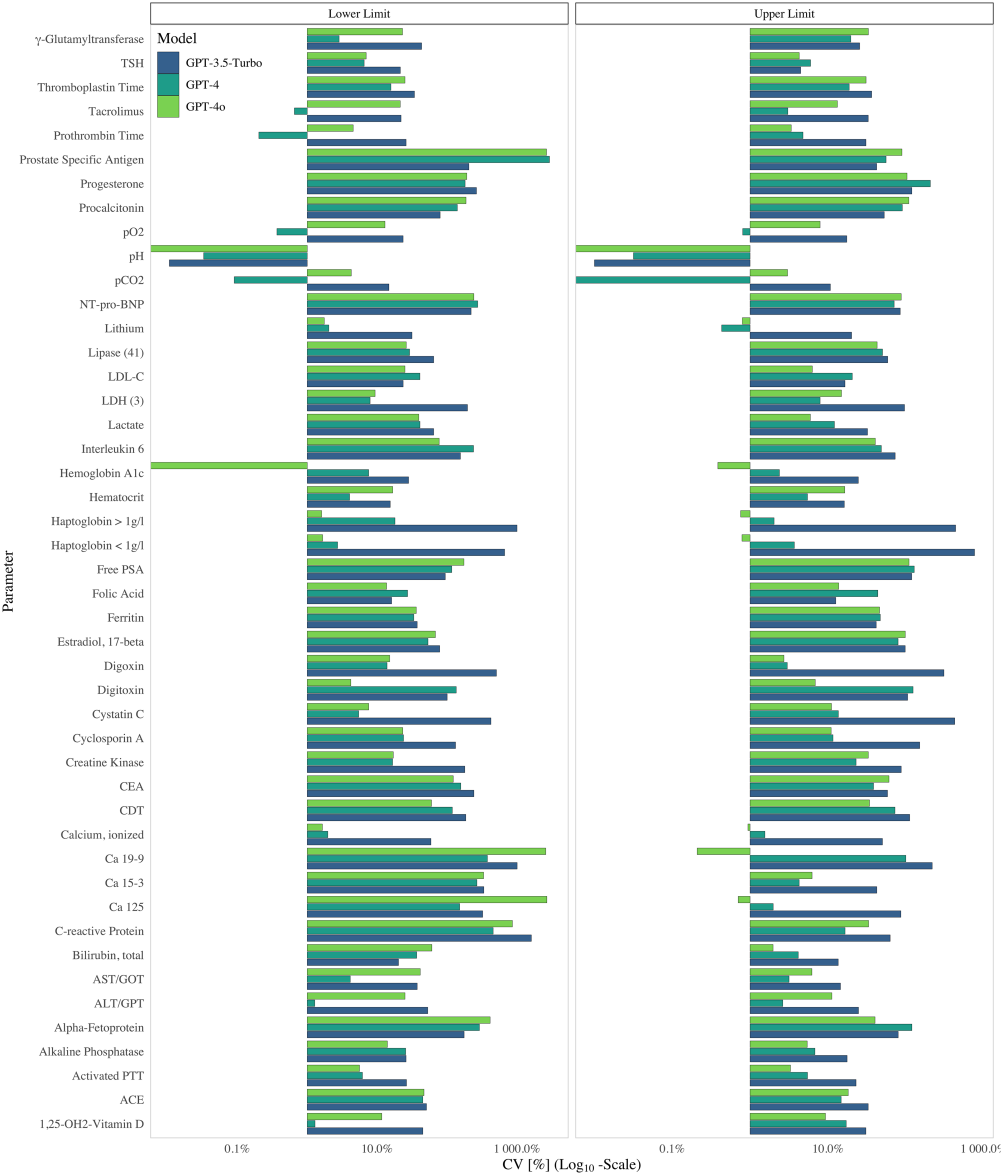
Coefficient of Variation (%) on a Log₁₀ Scale for Upper and Lower Limit of the measurands supplied by GPT-3.5-Turbo (blue) GPT-4 (green), GPT-4o (light green), 2024.

Analytical harmonization had a strong effect on variability. Analytes classified as “adequate/maintained” exhibited the lowest CVs, whereas those classified as ‘None/Needed’ showed CVs that were three times higher at the lower limit and over twice as high at the upper limit (*p* < 0.001; Figure 1; Appendix 2). Notably, measurands standardized to a limited extent like tumor markers such as free PSA or Ca 19–9 nearly consistently displayed such elevated CVs in contrary to well standardized analytes like pH (Figures 1, 2; Appendix 3).

**Figure 3:**
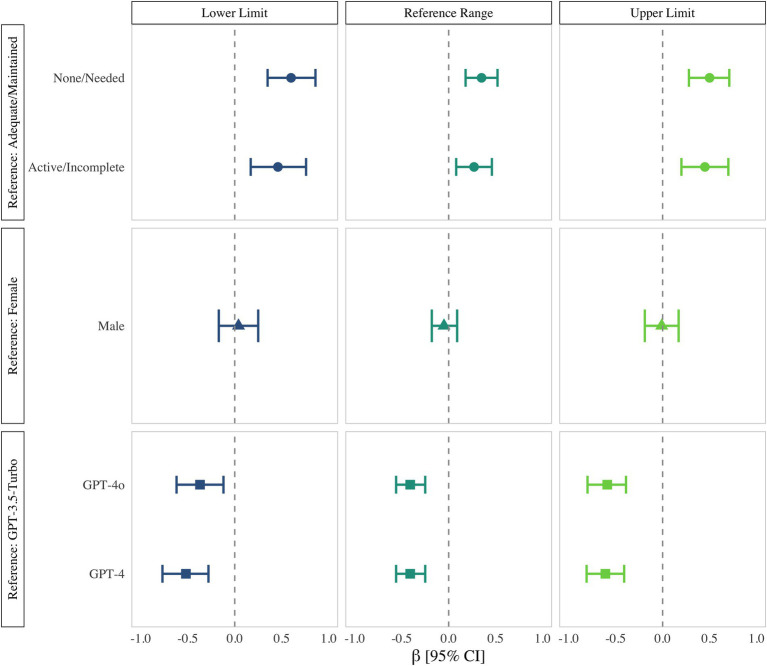
Univariate regression of the coefficient of variation for ChatGPT-derived laboratory reference intervals (*β*, log₁₀ scale; 95% CI). Marker shapes denote harmonization status (●), gender (▲), and model version (■), while lower limit is colored in blue, reference range in green, and upper limit in light green.

Univariate regression confirmed these patterns. Compared with GPT-3.5-Turbo, both GPT-4 and GPT-4o were independently associated with significantly lower CVs for the lower limit, upper limit, and reference range (all *p* < 0.001). Gender again showed no predictive value, whereas analytes lacking harmonization remained strong positive predictors of high variability (all *p* < 0.001) (Figure 3; Appendix 4).

## 4 Discussion

Despite their promising performance in medical licensing and certification exams globally (Meyer et al., 2024a; Liu et al., 2024), current AI-based chatbots, such as ChatGPT, demonstrate notable limitations in addressing laboratory medicine queries under real-world conditions (Meyer et al., 2024b). This issue is particularly pressing given the rising prevalence of self-diagnosis among ChatGPT users (Shahsavar and Choudhury, 2023). In such scenarios, patients often seek interpretations of laboratory results online, yet commonly neglect to provide the corresponding reference intervals (Meyer et al., 2024b)—a critical omission that compromises post-analytical accuracy (Sikaris, 2015).

### 4.1 High coefficients of variation in reference intervals

Our findings indicate, while iterative refinements in LLMs have led to notable improvements in repeatability and internal consistency – particularly in GPT-4o and GPT-4 compared to GPT-3.5-Turbo – relevant challenges remain. Although this trend aligns with previous observations of substantial performance gains achieved by GPT-4 over its predecessor in medical exams (Meyer et al., 2024a; Ibrahim et al., 2023; Yang et al., 2024), it also underscores ongoing concerns regarding repeatability, as reported in other clinical contexts (Franc et al., 2024; Franc et al., 2024).

Such persistent difficulties with repeatability, especially regarding reference intervals, are concerning, potentially leading to misinterpretation of results if patients or clinicians inadvertently rely on LLM-derived reference intervals. Variability of this magnitude exceeds typical allowable error thresholds used in clinical laboratory quality assurance, highlighting a fundamental misalignment between the probabilistic nature of LLM outputs and the precision required in laboratory medicine. Indeed, previous work has shown that incomplete prompts – those lacking reference intervals – can exacerbate post-analytical errors in LLM outputs (Meyer et al., 2024b). Ensuring that reference intervals are consistently included in prompts appears essential to mitigate these inaccuracies and reduce post-analytical errors (Nguyen et al., 2024; Wang et al., 2024).

Of particular concern are analytes that lack adequate or maintained harmonization. For these tests the chatbots consistently produced high coefficients of variation, plausibly because the models have comparatively little high-quality training data for such parameters. Therefore, analytes standardized to a limited extent not only confuse patients but also challenge LLMs (Jones and Barker, 2007). These findings underscore the urgency of international efforts to establish reference materials [International Federation of Clinical Chemistry and Laboratory Medicine (IFCC), 2024a] and to harmonize reference intervals, such as those led by the International Federation of Clinical Chemistry and Laboratory Medicine (Gillery and Young, 2013) or by working groups from Canada and Australia (Bohn et al., 2023; Koerbin et al., 2018). It is a well-known fact that the clinical interpretation of reference intervals and limits asks for harmonization (Klee, 2004) or if possible, for standardization. Thus, it is evident, that global standardization and harmonization is not merely a means of reducing data complexity (Vesper et al., 2016), thereby preventing comprehension problems and interpretation errors (Jones and Barker, 2007), but is also essential for enhancing the quality of AI-assisted tools in clinical laboratory medicine.

### 4.2 High variability in units

Moreover, the observed substantial variability in units, their notations and their spelling suggests that data heterogeneity extends beyond reference intervals to the units themselves. Notably, the analytes most affected by unit inconsistencies may also correspond to those for which LLMs are least reliable. Excluding such outputs to preserve analytic rigor may therefore have even led to an underestimation of real-world variability. These errors reflect the underlying heterogeneity within the LLM’s training corpus, where language variations and non-standard data introduce noise. Such inconsistencies exacerbate interpretive challenges, particularly for laypersons, and underscore the limitations of LLMs as “stochastic parrots”—systems driven by statistical correlations rather than causal understanding (Bender et al., 2021). When combined with the variability in reference intervals, these inconsistencies exacerbate interpretive challenges. Addressing this issue requires adherence to standardized units and notations, as recommended by the European Federation of Clinical Chemistry and Laboratory Medicine (Hansen, 2019) and underline the importance of harmonization the total testing process (Plebani, 2013; Plebani, 2016; Plebani, 2018). Implementing these recommendations within LLM training frameworks could thus further foster output consistency and usability in clinical contexts.

### 4.3 Limitations

Nevertheless, the nature of this study poses several limitations. This study focused on three versions of ChatGPT from a single provider (OpenAI). This scope reflects pragmatic resource constraints, as large-scale API access to other commercial LLMs (e.g., Gemini, Claude, Llama 3) would have required substantial funding. Nevertheless, ChatGPT represents not only one of the most widely used chatbot platforms worldwide, but its users also display a high tendency for self-diagnosis, making these findings clinically relevant. Moreover, the presented methodology is model-agnostic and can be applied to other systems in future collaborations or funded projects.

A further major limitation of this study is the use of a rigid, uniform prompt for a single hypothetical patient profile. While this approach enhanced internal consistency and allowed us to isolate model-intrinsic variability, it inevitably limited the assessment of the models’ contextual reasoning. More complex and clinically variable inputs – for instance, including age-related comorbidities such as renal insufficiency or hepatic dysfunction – could elicit different or even inconsistent adjustments of reference intervals. Thus, our design may have underestimated the variability that could occur in real-world, patient-specific queries, where models dynamically adapt to contextual cues. Future studies should systematically incorporate such contextual variability to better understand how patient- and prompt-related factors influence the reliability of LLM outputs.

Another important limitation concerns the unit-consistency threshold: by excluding analytes for which fewer than 80% of outputs used a consistent unit notation, we may have introduced a selection bias in regard to analytes. This criterion was implemented to ensure processability and avoid artificial inflation of variability metrics. However, it likely led to the preferential exclusion of analytes with intrinsically high unit variability. Future studies could mitigate this bias by applying unit-harmonization algorithms or stratifying analyses based on unit-consistency levels.

### 4.4 Implications for clinical practice

Despite these limitations, this study yields four important implications for the clinical use of AI-based chatbots in laboratory medicine.

Firstly, due to the inherent variability and potential for misinterpretation, such chatbots should be exclusively utilized by healthcare professionals who are trained to formulate high-quality prompts and able to critically assess the outputs. It is imperative to discourage patients from using chatbots to interpret their own laboratory results.

Secondly, the newer LLM versions tested, specifically GPT-4 and GPT-4o, demonstrate significantly lower variability compared to GPT-3.5-Turbo, making them preferable for future healthcare applications.

Thirdly, to further mitigate variability, adopting standardized prompt designs that consistently incorporate reference intervals is essential, as the absence of these safeguards will lead to persistent post-analytical errors.

Fourthly, concerted global initiatives for standardization or harmonization and the identification of reference measurement procedures and reference materials are important [International Federation of Clinical Chemistry and Laboratory Medicine (IFCC), 2024b]. Especially for LLMs it is of worth to have semantic and syntactic standards for laboratory reports available (Bietenbeck and Streichert, 2021). This is of course not limited to reference intervals and units but needs to be extended to Logical Observation Identifiers Names and Codes or laboratory reporting formats. Once standardization and harmonization are achieved, integrating these laboratory datasets into LLM training could enhance reproducibility, particularly for analytes that are currently poorly defined.

Finally, the misalignment between the precision required in laboratory medicine and the probabilistic nature of LLM outputs, combined with the high tendency of non-medical users to self-diagnose and omit reference intervals, creates a potentially hazardous situation. From a regulatory perspective, these findings underscore the importance of aligning LLM use in clinical contexts with emerging frameworks such as the EU Artificial Intelligence Act and FDA guidance on Software as a Medical Device. These frameworks emphasize transparency, risk management, and traceability—principles that are particularly relevant when outputs may influence clinical decision-making (Baumgartner and Baumgartner, 2023).

In summary, while advances in AI models hold promise for clinical laboratory medicine, relevant challenges remain in ensuring reliable and reproducible interpretations of laboratory data.

## 5 Conclusion

At present, ChatGPT’s high variability in regard to reference intervals leaves “curbside consultation” a mere aspiration for the future (Lee et al., 2023). Even after minimizing linguistic ambiguity (Yang et al., 2023) challenges such as hallucinations and output variability persist. Therefore, two practical implications remain. First, chatbots accessible to medical laypersons should be trained (or rule-augmented) to detect when a laboratory query lacks an explicit reference interval and prompt the user to supply it, rather than returning a possibly misleading value. Second, every gain in real-world reference interval harmonization and standardization will indirectly stabilize LLM outputs, because the models’ training corpora will contain fewer conflicting examples. Thus, while ongoing model refinement and domain-specific training are essential, they are not sufficient on their own. True progress depends on the global standardization and harmonization of reference intervals and units, as well as continued investigation into the capabilities and limitations of language models. Achieving these objectives will bridge the gap between current experimental applications and the reliable, real-world use of AI-driven chatbots in clinical decision support.

## Data Availability

The raw data supporting the conclusions of this article will be made available by the corresponding author on reasonable request.
